# Participation of Krüppel-like Factors in Atherogenesis

**DOI:** 10.3390/metabo13030448

**Published:** 2023-03-19

**Authors:** Stanislav Kotlyarov, Anna Kotlyarova

**Affiliations:** 1 Department of Nursing, Ryazan State Medical University, 390026 Ryazan, Russia; 2 Department of Pharmacy Management and Economics, Ryazan State Medical University, 390026 Ryazan, Russia

**Keywords:** Krüppel-like factors, atherosclerosis, endothelium, metabolism, immunometabolism, pathogenesis

## Abstract

Atherosclerosis is an important problem in modern medicine, the keys to understanding many aspects of which are still not available to clinicians. Atherosclerosis develops as a result of a complex chain of events in which many cells of the vascular wall and peripheral blood flow are involved. Endothelial cells, which line the vascular wall in a monolayer, play an important role in vascular biology. A growing body of evidence strengthens the understanding of the multifaceted functions of endothelial cells, which not only organize the barrier between blood flow and tissues but also act as regulators of hemodynamics and play an important role in regulating the function of other cells in the vascular wall. Krüppel-like factors (KLFs) perform several biological functions in various cells of the vascular wall. The large family of KLFs in humans includes 18 members, among which KLF2 and KLF4 are at the crossroads between endothelial cell mechanobiology and immunometabolism, which play important roles in both the normal vascular wall and atherosclerosis.

## 1. Introduction

Atherosclerosis is a global medical and social problem that is associated with a high prevalence of atherosclerotic cardiovascular diseases (ASCVD) [1,2]. ASCVD associated with atherosclerotic lesions of the coronary arteries, arteries of the lower extremities, and cerebral arteries makes a significant contribution to the structure of morbidity and mortality of the population [3,4]. Atherosclerosis carries a heavy economic and social burden both for individual patients and the economies of entire states [5,6,7]. Therefore, early diagnosis and prevention of these diseases is an important medical task [8]. In the uneasy history of the study of atherosclerosis, many hypotheses have been proposed, suggesting the role of various factors as a key cause of atherosclerosis development. Current understanding of atherogenesis suggests that the development of atherosclerosis is the result of a complex chain of events that occurs in the vascular wall involving various cells over many years and is driven by the complex effects of local and systemic factors, which include dyslipidemia, systemic inflammation, oxidative stress, and hemodynamic disturbances.

Hemodynamic forces, such as shear stress, are the most important factors determining endothelial function. Endothelial cells monolayer the entire vascular bed and form a barrier between blood and tissues. This barrier ensures the maintenance of the necessary blood pressure transport of substances and cells. The endothelium is actively involved in the regulation of blood flow, producing various substances that promote both vasodilation and vasoconstriction [9]. This very important function can be impaired by endothelial dysfunction, which is the subject of numerous studies in vascular biology [10]. The ability of the endothelium to detect and respond to hemodynamic changes is complex in nature and involves various mechanisms that can be impaired in atherosclerosis [11]. It is important to note that under normal physiological conditions, laminar shear stress promotes the production of nitric oxide and other vasoactive substances that help maintain vascular homeostasis. However, disruption of flow patterns, such as low or oscillatory shear, can activate endothelial cells and promote proinflammatory and prothrombotic states, leading to the recruitment of immune cells and initiation of atherogenesis.

The innate immune system plays a critical role in the initiation and progression of atherosclerosis. Immune cells, such as monocytes, are key participants in atherogenesis because of their role in the uptake of atherogenic lipid fractions and participation in innate immune responses in the vascular wall [12]. These cells are recruited into the vascular wall from the peripheral bloodstream, where they differentiate into macrophages [13]. Macrophages are not a homogeneous cell type. On the contrary, macrophages have polarized phenotypes based on cell metabolic features that are cross-linked with their immune function [14,15]. Cellular metabolism plays a crucial role in maintaining cellular homeostasis and energy production. It has been shown that the metabolic state of immune cells, such as macrophages, can modulate their function and phenotype, which has implications for atherogenesis.

A growing body of evidence strengthens the understanding of the relationship between hemodynamics, cellular metabolism, and the innate immune system in the pathogenesis of atherosclerosis. Flow disorders may alter the metabolic state of endothelial cells and contribute to the production of proinflammatory cytokines, which may activate immune cells and promote atherogenesis. In addition, activation of innate immune receptors can trigger proinflammatory signaling pathways and alter the metabolic state of immune cells, leading to the development of proatherogenic phenotypes. These processes have complex regulation. Krüppel-like factors (KLF) are a family of transcription factors that play an important role in regulating the expression of various genes, due to which they are involved in the regulation of many cellular functions and pathways, such as cell proliferation, apoptosis, inflammation, and lipid metabolism. KLFs, among which KLF2 and KLF4 are of the highest research and clinical interest, are expressed in various cells in the vascular wall and are involved in the regulation of many biological processes that are important for normal vascular function and that may be impaired in atherogenesis.

Thus, the aim of this review is to discuss the role of KLF in the regulation of metabolic and immune mechanisms in atherogenesis.

## 2. The Biological Function of Kruppel-like Factors

KLFs are a family of zinc-finger transcription factors with important cellular functions ranging from involvement in embryonic stem cell differentiation, cell proliferation, cell differentiation, and metabolism to apoptosis (Table 1) [16,17,18,19]. Currently, 18 members of the KLF family are known and are characterized by a wide range of expression in many tissues and organs, such as the liver, heart, adipose tissue, lungs, myeloid cells, skeletal muscle, etc. [19]. Changes in their expression lead to the dysregulation of metabolic processes that are associated with several human diseases [19]. KLFs play an important role in the regulation of endothelial function and other biological processes in the vascular wall in the norm and in various diseases, such as atherosclerosis [20].

KLFs are expressed in many animal and human species, as well as in unicellular organisms [18]. They are homologous in nature and structure to the Krüppel proteins of *Drosophila melanogaster*, which regulate body segmentation during fly embryogenesis [21,22,23]. They also got their name from this protein Krüppel, which in German means “cripple” (“Kruppel”). This protein in Drosophila is responsible for the development of fly larvae [19].

KLFs have two main domains in their structure: the carboxy-terminal region and the N-terminal region. The carboxy-terminal region is conserved, having three C2H2 zinc finger domains closer to the C-terminus [18,24]. Because of this, they regulate transcriptional activation [25,26]. The C2H2 zinc finger domain contains two cysteine and two histidine residues, which are coordinated by the zinc ion [18,27,28]. The zinc finger factors bind to Guanine-Cytosine-rich regions and CACC elements (GT boxes) and act as activators or repressors of transcription [19,29]. N-terminal regions are less conservative and very variable. They contain additional different combinations of transactivation/repressor domains. Their main function is participation in protein-protein and protein-DNA interactions. Some KLFs additionally have nuclear localization signals (NLS) (characteristic of KLF1, KLF2, KLF4, KLF6, KLF8, KLF15) and nuclear export signals (NES) (characteristic of KLF5 and KLF6), which regulate their location inside the cell.

Traditionally, KLFs are divided into three groups based on the domain architecture: KLF3, 8, and 12 groups; KLF1, 2, 4, 5, 6, and 7 groups; and KLF9, 10, 11, 13, 14, and 16 groups [30]. KLFs belonging to the first group act as transcriptional repressors. Members of the second group activate the transcription of dependent genes, while members of the third group promote both activation and repression of the transcription of dependent genes [30,31].

KLFs are associated with the functioning of various processes, but in this review, we focused on the role of KLFs in the regulation of metabolic and immune homeostasis in predominantly endothelial cells and their connections with other cells of the vascular wall during atherogenesis.

### Immune and Metabolic Functions of KLF Family Members

KLF2 is the most studied transcription factor because of the importance of its functions and involvement in many metabolic processes. KLF2 is critical for blood vessel development during the embryonic stage. KLF2 knockout mice have impaired blood vessel maturation, leading to fatal embryonic heart failure [32,33].

KLF2 is expressed mainly in endothelial cells and is a central regulator of gene expression and endothelial function [34]. In addition to the endothelium, KLF2 expression is characteristic of many cells of the immune system, such as monocytes, B cells, and T cells, including CD4+ (helper) and CD8+ (killer) cells [31,35,36]. In endothelial cells and monocytes, proinflammatory stimuli such as interleukin (IL)-1β, Tumor necrosis factor-alpha (TNFα), and lipopolysaccharides (LPS) inhibit KLF2 expression. At the same time, KLF2 also suppresses LPS-dependent monocyte activation and expression of cyclooxygenase-2 (COX2), IL-1β, IL-8, TNFα, macrophage inflammatory protein-1 alpha (MIP-1α), and Monocyte Chemoattractant Protein 1 (MCP-1) [36,37]. KLF2 levels in monocytic cells are reduced during their differentiation and transformation into macrophages [38].

KLF2 plays a crucial role in the molecular mechanisms of atherosclerosis, regulating multiple pathways involved in endothelial cell function, inflammation, and lipid metabolism. The protective effects of KLF2 in atherosclerosis make it an attractive target for the development of new treatments for cardiovascular disease.

The KLF4 factor has a similar structure and function to KLF2, and of all members of the KLF family, it is the most similar to KLF2 [39]. Expression of KLF4 is similar in tissue specificity to KLF2 and is characteristic of tissues such as vascular endothelium, lymphoid cells, skin epithelial cells, intestine, kidney, and lung [30,40,41]. Like KLF2, KLF4 is activated by shear stress in endothelial cells [39,42,43]. These transcription factors are more highly expressed in straight vessel segments and decreased at branch sites, which are areas of the vascular network more susceptible to atherosclerosis [44,45,46,47]. KLF4 is also involved in the functions of cells other than endothelial cells. It has been shown that KLF4 may be involved in the regulation of myeloid differentiation, and KLF4 deficiency may reduce the number of monocytes and possibly shift the differentiation of primary common myeloid progenitors (CMPs) toward granulocytes [48]. KLF4 overexpression in B cells resulted in decreased c-Myc expression [49].

In endothelial cells, KLF5 has been shown to play a pathogenic role in the development of atherosclerosis [50,51]. KLF5 plays an important role in the proliferation of vascular smooth muscle cells (VSMCs) and is induced in response to vascular injury. The mechanism by which KLF5 is induced in proliferating neointimal VSMCs in response to vascular injury is complex and involves several signaling pathways; KLF5 activates promoters of several genes, such as plasminogen activator inhibitor-1 (PAI-1), inducible nitric oxide synthase (iNOS), platelet-derived growth factor subunit A (PDGF-A), Early growth response protein 1 (Egr-1), PI3K/Akt, mitogen-activated protein kinase (MAPK), and vascular endothelial growth factor (VEGF) receptors, affecting the cell cycle and other processes [52,53,54].

Vascular damage has been shown to result in the release of growth factors, such as PDGF and fibroblast growth factor (FGF), from platelets and cells of damaged tissues [55,56,57]. These growth factors activate receptor tyrosine kinases (RTKs) on the surface of VSMCs, leading to the activation of downstream signaling pathways [56,58,59].

In turn, RTKs activate two major signaling pathways in VSMCs, PI3K/Akt and MAPK [60,61,62,63]. These pathways play an important role in cell proliferation, survival, and differentiation. Activation of these pathways leads to phosphorylation and activation of several transcription factors, including KLF5 [64,65,66,67]. Activation of the PI3K/Akt and MAPK pathways leads to increased KLF5 gene expression in VSMCs. KLF5 mRNA levels increase rapidly in neointimal VSMCs after vascular injury [50,68,69].

KLF5 mRNA is translated into the KLF5 protein in the VSMCs cytoplasm. The newly synthesized KLF5 protein is then transported to the nucleus. Once in the nucleus, KLF5 binds to specific DNA sequences in the promoters of target genes, leading to their transcriptional activation. KLF5 regulates the expression of several genes involved in cell cycle progression, including cyclin D1 and cyclin-dependent kinase 4 (CDK4) [64,70,71,72]. Activation of KLF5 and its downstream target genes promote VSMCs proliferation and cell cycle progression, leading to neointima formation and vascular remodeling.

The relationship between KLF5 and miRNA-576 also plays an important role in the pathogenesis of atherosclerosis and contributes to disease progression. Overexpression of miRNA-576 suppressed KLF5 and β-catenin [73].

KLF5 expression is increased in endothelial cells in response to various proatherogenic stimuli, such as impaired blood flow and high levels of low-density lipoprotein (LDL) cholesterol. Overexpression of KLF5 in endothelial cells contributes to endothelial dysfunction, oxidative stress, and inflammation, which are key hallmarks of atherosclerosis. KLF5 can contribute to endothelial dysfunction by regulating the expression of genes involved in vascular tone, permeability, and inflammation. For example, KLF5 can increase the expression of endothelin-1, a potent vasoconstrictor [74,75], and decrease the expression of eNOS, which produces the vasodilator nitric oxide [76,77]. KLF5 can also increase the expression of vascular cell adhesion molecule 1 (VCAM-1), C-X-C Motif Chemokine Ligand 1 (CXCL1), matrix metallopeptidase 9 (MMP9), VEGF, and other genes [78,79,80] that directly play a key role in many processes, including leukocyte adhesion and migration, resulting in increased vascular permeability and inflammation. For example, KLF5 binds directly to the VEGF promoter and induces transcription and protein formation. A decrease in KLF5 attenuates VEGF mRNA expression and protein secretion [81,82].

In addition, KLF5 can contribute to oxidative stress by regulating the expression of genes involved in reactive oxygen species (ROS) production and detoxification [74,83]. KLF5 can upregulate the expression of NADPH oxidase subunits [83] that produce ROS and downregulate the expression of antioxidant enzymes such as SOD and catalase [83]. This dysregulation can lead to an imbalance between ROS production and detoxification, resulting in increased oxidative stress.

KLF5 can promote inflammation by regulating the expression of genes involved in the innate immune response [20,77]. KLF5 can increase the expression of cytokines such as IL-1, IL-6, and TNF-α and adhesion molecules. This can lead to the production of additional ROS and proinflammatory mediators, supporting the cycle of oxidative stress and inflammation [82,84].

KLF5 contributes to the phenotypic switch of smooth muscle cells from a contractile to a synthetic phenotype, which is associated with the development of atherosclerosis [82,85]. KLF5 overexpression increases the ability of macrophages to migrate and proliferate [20,86]. KLF5 is stabilized from degradation by TNF-α because TNF-α promotes the sumoylation of KLF5 while decreasing ubiquitination [20,86,87]. This process helps KLF5 remain active in response to inflammatory stimuli.

Another member of the KLF family, KLF6, regulates several genes involved in the development of atherosclerosis, including genes involved in inflammation, lipid metabolism, and vascular smooth muscle cell proliferation. In addition, KLF6 activates transforming growth factor (TGF) β, TGFβ receptors, TGFβ-stimulated genes, and urokinase plasminogen activator [45,52].

KLF6 expression is induced in smooth muscle cells in response to vascular damage [52]. In macrophages, KLF6 has been shown to play a protective role in the development of atherosclerosis. KLF6 responds strongly to proinflammatory macrophage stimuli [88]. Its expression is increased in macrophages stimulated by factors promoting their M1 polarization but decreased by factors stimulating M2 polarization [20,88]. Thus, KLF6 plays an important role in the regulation of gene transcription for macrophage M1 and M2 polarization [89]. In particular, KLF6 is required for optimal binding of p65 to its target gene promoters, and it promotes nuclear factor kappa B (NF-κB)-targeted transcription by interacting with p65 [20,90]. In addition, KLF6 suppresses B-cell lymphoma 6 (BCL6) expression, resulting in increased proinflammatory gene expression and enhanced macrophage motility [91,92]. On the other hand, KLF6 binds to PPARγ and suppresses its ability to induce M2 gene transcription [88]. These data suggest that KLF6 is a dynamic regulator of macrophage polarization, playing a complex role in stimulating or suppressing inflammation depending on the cellular context.

Thus, KLFs exhibit a diversity of functions that may be of great clinical significance. That said, KLF2 and KLF4 are involved in many processes that occur in the vascular wall during atherogenesis. They are involved in the cross-links between hemodynamic disorders, lipid metabolism, and inflammation, i.e., key processes that are associated with the development of atherosclerosis. Given the importance of these processes for the normal vascular wall and atherogenesis, KLF2 and KLF4 are of growing research and clinical interest. They are considered regulators of these cross-links and cellular functions and appear to be promising targets for the development of new diagnostic and therapeutic agents.

## 3. Role of KLF2 and KLF4 in Cross-Links of Cellular Immunometabolism, Hemodynamics, and Atherogenesis

### 3.1. Regulation of Hemodynamics and Angiogenesis

Endothelial cells line the inner surface of blood vessels and are constantly exposed to hemodynamic forces arising from blood flow. The hemodynamic conditions to which endothelial cells are exposed can affect their structure and function. Shear stress is an important hemodynamic factor affecting endothelial function [93,94]. Shear stress regulates the expression of a number of genes involved in endothelial function, including genes involved in inflammation, angiogenesis, and coagulation. In addition, shear stress also regulates the endothelial cell cytoskeleton. The cytoskeleton is represented by a dynamic network of protein filaments that provides structural support and plays a key role in cell migration, adhesion, and signal transduction. Shear stress has been shown to regulate the organization and dynamics of the cytoskeleton in endothelial cells [95,96]. In response to shear stress, endothelial cells undergo significant cytoskeleton rearrangements, including reorientation and elongation of actin filaments [97,98]. This contributes to the polarization of endothelial cells under shear stress. Polarization of endothelial cells involves the establishment of asymmetric distribution of organelles and proteins along the apical-basal axis of the cell [99,100]. On the contrary, in the sections with the impaired flow, to which a low shear stress corresponds, the cells do not have a polarized shape. Such sections of arteries, which correspond to areas of curvatures and bifurcations, are more prone to atherogenesis [94,101].

A growing body of evidence suggests cross-linkages between endothelial cell metabolism and function [102]. Changes in glucose levels have been shown to affect the orientation of endothelial cells in the brain microcirculatory bed, including the orientation of cell nuclei and F-actin [103]. The disintegration of the cytoskeletal structure of actin filaments in energy-depleted endothelial cells has been shown previously [104]. Thus, glycolysis may be an important source of energy for cytoskeleton remodeling. The activity of glycolysis may be related to the regulation of the cytoskeleton, orientation, and polarization of endothelial cells. Inhibition of glycolysis in endothelial cells can disrupt apical-basal axis formation and disrupt actin cytoskeleton organization. It was shown that the glycolytic enzyme aldolase is involved in the formation of F-actin bundles through the stabilization of parallel F-actin filaments, which leads to the formation of a less organized branched F-actin cytoskeleton [103,105]. At the same time, a higher level of glycolysis contributes to a greater involvement of aldolase in this process instead of F-actin stabilization, which leads to an increased migration ability of cells [103,105]. In addition, the enzyme 6-phosphofructo-2-kinase/fructose-2,6-bisphosphatase 3 (PFKFB3), which is a key regulator of glycolysis, plays a role in endothelial cell polarization and also promotes the expression of adhesion molecules such as Intercellular Adhesion Molecule 1 (ICAM-1), VCAM-1, and E-selectin [106,107]. In addition, PFKFB3 increases VE-cadherin levels in the plasma membrane [107]. PFKFB3 is also involved in endothelial cell migration and tube formation. Suppression of PFKFB3 impairs lamellipodia formation and impairs Tip cell activity, which is related to PFKFB3 participation in the control of filopodia/lamellipodia formation, which is partially mediated by its compartmentalization with F-actin in mobile protrusions [108].

As noted above, KLF2 is activated by laminar shear stress and regulates a wide range of endothelial cell functions, including proliferation, migration, and inflammation [46,109]. Overall, KLF2 is involved in the regulation of vascular hemodynamic forces and has a protective effect on the endothelium [109,110]. While KLF2 is expressed at high levels in endothelial cells in the area of laminar flow, its expression drops to low levels near the sites of blood flow disturbances, which are characterized by the development of atherosclerosis [46].

KLF2 overexpression was shown to reproduce the inhibitory effect of laminar flow on endothelial glycolysis. Shear stress acting through KLF2 reduced the expression of key glycolytic enzymes such as PFKFB3 [111]. KLF2 has been shown to suppress PFKFB3 promoter activity so that shear stress-mediated repression of endothelial cell metabolism controls their phenotype [111]. Thus, laminar shear stress reduces PFKFB3 expression, which leads to a decrease in glycolysis. On the other hand, low shear stress and oscillatory flow can promote endothelial dysfunction, promoting a switch to glycolytic metabolism. High glucose levels are also relevant. Reduced KLF2 expression may exacerbate endothelial damage in diabetic nephropathy. In cell culture, it has been shown that KLF2 expression was suppressed with increasing glucose levels, whereas insulin, in contrast, led to increased KLF2 expression [110]. At the same time, partial and temporary blockade of PFKFB3 leads to a decrease in glycolysis and reduces pathological angiogenesis [112].

In addition, KLF2 has been shown to regulate the function of several cytoskeletal proteins. KLF2 regulates the phosphorylation and activity of cytoskeleton-related F-actin proteins [113]. Shear stress promotes actin cytoskeleton remodeling through activation of the MEK5/ERK5/MEF2 MAPK pathway, which transcriptionally induces KLF2. KLF2 indirectly activates RhoA, which induces the formation of actin shear fibers that are necessary for both alignment in the flow direction and inhibition of the FAK-JNK-c-Jun/ATF2 signaling pathway [113]. Thus, KLF2 promotes cell alignment in the flow direction through the formation of a thick network of actin shear fibers that is very different from classical tension fibers [113].

KLF2 may also affect hemodynamics because it plays an important role in the stress-induced shear regulation of endothelial nitric oxide synthase (eNOS) [114]. eNOS is an important regulator of hemodynamics through the production of nitric oxide (NO), which is considered a key player in the regulation of vascular tone through its vasorelaxant capacity. Reduced bioavailability of eNOS-derived NO is considered as a critical step in endothelial dysfunction and atherogenesis. Studies have shown that KLF2 induces eNOS expression and increases NO production in cultured human umbilical vein endothelial cells (HUVECs) [115]. It is assumed that KLF2 promotes NO production by regulating eNOS uncoupling via Nrf2 (Nuclear factor erythroid 2-related factor 2)/ HO-1 (heme oxygenase-1) [115]. Thus, activation of KLF2 in endothelial cells causes induction of eNOS and has a vasodilatory effect.

In turn, NRF2 regulates glycolysis and cell proliferation by regulating transcription of KLF2, PFKFB3, VEGFA, forkhead box protein O1 (FOXO1), and MYC, thereby controlling the switch between the active and resting states of the endothelium [116]. This is because NRF2 is a regulator of miR-93 expression that targets both KLF2 and PFKFB3, which act as inhibitors and stimulators of glycolysis, respectively. This microRNA also acts on FOXO1, VEGFA, and MYC, which regulate cell proliferation [108,111,116,117]. NRF2 has also been shown to be a known mediator of oxidized 1-palmitoyl-2-arachidonoyl-sn-glycero-3-phosphocholine (oxPAPC) effects, which are produced by exposure of active oxygen species to PAPC, which is a component of cell membranes [116]. It was shown that oxPAPC NRF2-dependently induces glycolysis and proliferation in endothelial cells. At the same time, endothelial cells [116] are a significant source of extracellular miR-93, which are found in the exosomal fraction [116].

KLF2 has a potent anti-angiogenic effect. KLF2 has been shown to induce the expression of genes that promote cell cycle arrest and inhibit cell proliferation, presumably by affecting the VEGF-mediated pathway. The molecular mechanism is the ability of KLF2 to inhibit VEGFR2/KDR expression [118]. KLF2 also attenuates cell migration by affecting multiple genes, including VEGFR2 and SEMA3F (Semaphorin 3F) [114]. Thus, when KLF2 levels in cells are increased, VEGFR2 levels are decreased [114]. In general, KLF2 helps to maintain the integrity of the endothelial monolayer and prevent the formation of atherosclerotic lesions [32].

KLF2 has also been shown to affect angiogenesis through the inhibition of hypoxia-inducible factor 1-alpha (HIF-1α) by promoting HIF-1α degradation, affecting its folding and maturation [119]. KLF2 overexpression was shown to significantly inhibit hypoxia-induced endothelial tube formation, whereas KLF2 deficiency promotes hypoxia-mediated angiogenesis in vivo [119].

Another member of the KLF family, KLF4, has many similar functions as KLF2. KLF4 plays a key role in the regulation of the endothelial cell cytoskeleton. KLF4 has been shown to regulate the function of several cytoskeletal proteins [120]. In addition to the regulation of cytoskeletal proteins, KLF4 also regulates the expression of proteins involved in the formation and maintenance of endothelial cell junctions. Endothelial cell junctions are crucial for maintaining endothelial integrity and regulating vascular tone and blood flow. KLF4 has been shown to regulate the expression of several functional proteins, including VE-cadherin [121]. At the same time, Klf4 knockdown disrupted the endothelial barrier and also enhanced lipopolysaccharide-induced lung injury and pulmonary edema in mice [121].

In addition, KLF4 is also involved in other hemodynamic mechanisms, including eNOS regulation [122,123]. At the same time, vasorelaxation in pulmonary arteries has been shown to be impaired under nitrosative stress, which is associated with S-nitrosation of KLF4. At the same time, endothelin-1 stimulated S-nitrosylation of KLF4 in endothelial cells [123]. These findings are of interest because of the role of smoking in the pathogenesis of atherosclerosis and chronic obstructive pulmonary disease and the role of nitrosative stress in their development.

Thus, KLF2 and KLF4 are important participants in hemodynamic regulation, and this function has close cross-linkages with other endothelial cell functions.

### 3.2. Immunometabolism of Endothelial Cells

In addition to the fact that endothelial cells play an important role in vascular functions such as vasodilation, blood pressure regulation, and angiogenesis, a growing body of evidence is increasing the understanding that endothelial cells are also involved in immune responses [124,125]. Endothelial cell function also includes the regulation of immune cell migration across the vascular barrier through the production of immune mediators such as cytokines and chemokines. Endothelial cells express several classes of molecules, such as selectins and integrins, which are involved in leukocyte adhesion and extravasation from the bloodstream. Endothelial cells may also themselves undergo an inflammatory response with activation of adhesion molecules and secretion of proinflammatory cytokines in response to stimuli such as LPS or other pathogens [125,126]. Endothelial cell activation is accompanied by a change in their metabolism, which is known as cellular immunometabolism. The significance of cellular immunometabolism is well known in the example of macrophage polarization. Macrophages are known to exhibit a differentiated role in inflammation. In addition to pro-inflammatory, “classically activated” M1 macrophages, “alternatively activated” M2 macrophages are known to contribute to the resolution of inflammation. Switching cellular metabolism to glycolysis is an important step in the M1 activation of macrophages.

Glycolysis is the main source of energy for endothelial cells [102,127,128,129]. Although endothelial cells have good access to oxygen from the blood, these cells produce up to 85% of ATP as a result of glycolysis rather than oxidative phosphorylation. Thus, under conditions of migration, proliferation, or stress, endothelial cells increase glycolysis to meet energy requirements [129,130,131]. Increased glycolysis plays an important role in the immunometabolism of endothelial cells, providing them with energy and metabolites for the immune response.

High levels of glycolysis in endothelial cells are associated with the regulation of several rate-limiting stages, which are related to the action of hexokinase 2 (HK2) and phosphofructokinase 1 (PFK1). Meanwhile, PFK1 activity is controlled by PFKFB3, which produces fructose-2,6-bisphosphate, the main allosteric activator of PFK1 [132,133]. Glycolysis in endothelial cells is regulated by various signaling pathways, including the PI3K/Akt and MAPK pathways [134]. These pathways are activated in response to proinflammatory signals that activate glycolytic enzymes such as hexokinase and phosphofructokinase.

PFKFB3 plays an important role in the regulation of glycolysis, especially under pathological conditions and during angiogenesis [108,112,135,136]. In an experiment with primary human pulmonary artery endothelial cells and aortic endothelial cells that were stimulated with TNFα or LPS, or TNFα and LPS were shown to increase glucose uptake 2.2-fold, glycolysis 2.3-fold, and mitochondrial respiration 1.6-fold. The increase in glycolysis correlates with a 2-4-fold activation of PFKFB3 [111,137]. At the same time, TNFα and LPS were shown to stimulate the expression of mRNA and the KLF4 protein, which acts as a repressor of PFKFB3 transcription. KLF4 was also found to suppress inflammation by normalizing PFKFB3-mediated enhancement of glycolysis, and the KLF4-PFKFB3 signaling axis integrates immunometabolism in human arterial endothelial cells [111,137].

In addition, glycolysis is also regulated by transcription factors such as HIF-1α and c-Myc [138,139]. HIF-1α is activated under conditions of hypoxia, which is characteristic of the inflammatory environment and activates glycolytic enzymes, promoting ATP production [134]. Reduced c-Myc expression in endothelial cells leads to a proinflammatory senescent phenotype characterized by an enhanced inflammatory response and endothelial dysfunction [140].

KLF2 also modulates the inflammatory response in atherosclerosis [141]. KLF2 has been shown to suppress the expression of proinflammatory genes such as VCAM-1 and MCP-1 and promote the expression of anti-inflammatory genes such as eNOS and HO-1 [34,114,141,142,143,144,145,146]. This anti-inflammatory effect helps prevent the migration of monocytes and other inflammatory cells to the vessel wall and limits the development of atherosclerotic lesions.

Thus, KLF2 plays an important role in the regulation of proinflammatory activation of both endothelial cells and monocytes [31]. The anti-inflammatory effect of KLF2 has been shown to be related to the inhibition of monocyte proinflammatory gene expression, which inhibited the expression of several cytokines/chemokines such as IL-1β, IL-8, TNFα, MCP-1, and inflammatory factors, including tissue factor and COX-2 [145]. KLF2 exerts its anti-inflammatory effect through a decrease in the pro-inflammatory activity of NF-κB [145]. On the other hand, NF-κB inhibits KLF2 expression by interrupting the binding of MADS box transcription enhancer factor 2 (MEF2) and histone deacetylase (HDAC) molecule access to the KLF2 promoter [31]. Thus, KLF2 and NF-κB interact to regulate inflammatory pathways [31]. Interestingly, however, the consumption of fatty foods decreases myeloid KLF2 levels, which represents a mechanism for cross-linking risk factors in atherogenesis [147].

The role of KLF4 in atherosclerosis is complex and cell-type-dependent. KLF4 may play a protective role in endothelium but a pathogenic role in smooth muscle cells. Overexpression of both Klf4 and KLF2 has been shown to increase the expression of several anti-inflammatory and antithrombotic factors in endothelial cells [45]. Klf4 also inhibits TNF-α-induced expression of Vcam1 by blocking the binding of nuclear factor-κB to the Vcam1 promoter [148]. Interestingly, statins increase KLF4 expression, with KLF4 mediating the suppressive effect of statins on TNF-α-induced VCAM1 expression by reducing NF-κB binding to the VCAM1 promoter [149].

Meanwhile, KLF4 plays a key role in the proliferation and differentiation of VSMCs, acting as a “molecular switch” of their function [52]. Phenotypic switching and proliferation of VSMCs is an important part of atherogenesis. It was previously shown that KLF4 knockout mice exhibit enhanced neointima formation compared to control mice [150]. In this case, KLF4 acts as a suppressor of VSMCs differentiation and also as an inhibitor of proliferation of these cells in response to vascular injury [52]. Klf4 suppresses the proliferation of smooth muscle cells as well as the expression of numerous markers of their differentiation, such as smooth muscle (SM) 22α and SM α-actin [148,151]. On the other hand, cholesterol-loaded smooth muscle cells can induce KLF4 expression and transform smooth muscle cells into a foam cell phenotype, which is a key feature of atherosclerosis [152].

KLF4 has an anti-inflammatory effect on myeloid cells, whereas KLF4 deficiency in myeloid tissue leads to increased inflammatory cellular infiltration of atherosclerotic lesions [153]. KLF4 overexpression in macrophages has been shown to promote an anti-inflammatory M2 phenotype through transcriptional interactions with STAT6, whereas KLF4 deficiency in macrophages promotes a proinflammatory M1 phenotype [39,154,155,156].

**Figure 1:**
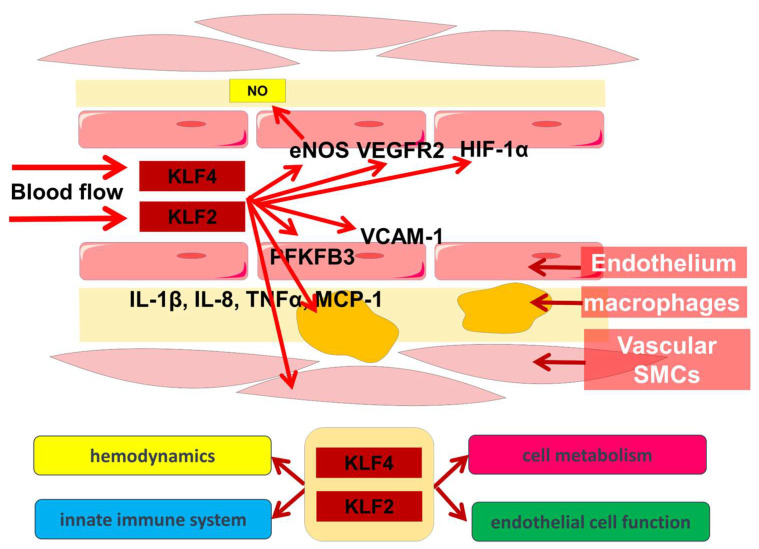
Involvement of KLF2 and KLF4 in hemodynamic, metabolic, and innate immune system cross-links.

Thus, metabolic and immune processes are closely cross-linked, with KLF2 and KLF4 playing an important role in their regulation (Figure 1).

### 3.3. Participation of KLF2 and KLF4 in Other Biological Processes in the Vascular Wall

Thus, KLF2 has a role in protecting the endothelium from inflammation and damaging factors, which plays an important role in preventing atherosclerosis [157,158]. In addition, KLF2 has been shown to regulate the expression of genes involved in lipid metabolism and transport, which are important for the development of atherosclerosis [159]. KLF2 has been shown to promote the expression of genes involved in cholesterol efflux, such as ATP binding cassette subfamily A member 1 (ABCA1) and ATP binding cassette subfamily G member 1 (ABCG1), which help remove excess cholesterol from macrophages and prevent the formation of foam cells, a hallmark of atherosclerosis. In addition, KLF2 deficiency is involved in the formation of primary macrophage foam cells through the potential regulation of the key protein FABP4 (fatty-acid binding protein 4), which transports fatty acids and is expressed in adipocytes and macrophages [146]. KLF2 has been shown to inhibit monocyte activation and phagocytic capacity [145]. At the same time, KLF2 expression in circulating monocytes is reduced in patients with coronary heart disease [145]. This is consistent with the evidence of increased lipid uptake and aP2/ FABP4 expression in KLF2-deficient hemizygous macrophages. Increased lipid uptake by macrophages may be responsible for increased atherosclerosis [146]. The results of these studies suggest that increasing KLF2 expression may be a novel strategy for preventing and treating atherosclerosis [160].

KLF4 increases the levels of cholesterol-25-hydroxylase and liver receptor X in endothelial cells and macrophages, which promotes an atheroprotective synergistic effect between these cells. KLF4-mediated transactivation of cholesterol-25-hydroxylase and hepatic X receptor promotes cholesterol efflux and M1-to-M2 conversion in macrophages [161]. These data reinforce the understanding that KLF2 and KLF4 have functional overlaps in myeloid and endothelial cells, demonstrating a similar evolutionary trajectory [39].

Of interest are the roles of microRNAs in KLF2 and KLF4 function and the overlap between lipid metabolism and hemodynamics. MiR-92a is involved in the regulation of KLF2 and KLF4 in endothelial cells [34,45,114,162,163]. The expression of miR-92a is increased in endothelial cells and blood flow in atherosclerosis. It has been shown that in turbulent blood flow, there is an increase in miR-92a expression, while in laminar flow, there is a decrease. Inhibition of miR-92a prevents endothelial dysfunction and atherosclerosis in mice [164].

This is because, acting through KLF2 and KLF4, miR-92a regulates endothelial cell activation by oxLDL under low-shear stress conditions [164]. Inhibition of miR-92a was shown to reduce ICAM-1 expression when exposed to low shear voltage both in the presence and absence of oxLDL. At the same time, miR-92a overexpression caused a significant increase in ICAM-1 expression [164].

In addition, miR-92a controls angiogenesis and functional repair of ischemic tissues by acting through integrin Subunit Alpha 5 (ITGA5) mRNA [165]. Downregulation of eNOS in response to miR-92a overexpression has also been shown to occur secondary to ITGA5 mRNA degradation [165].

Importantly, endothelial miR-92a is transported into macrophages mainly via extracellular vesicles, where it contributes to macrophage inflammatory activation [166]. miR-92a can also bind to high-density lipoprotein (HDL) in the serum of patients with coronary heart disease (CHD) [167]. In patients with CHD, miR-92a levels were significantly elevated, with endothelial cells being the main source of cells for miR-92-containing microvesicles. miR-92a transport by microvesicles is considered to be an important mechanism of angiogenesis regulation and a tool of intercellular communication [168]. Transported into macrophages mainly through extracellular vesicles, miR-92a regulates KLF4 levels, contributing to their atherogenic phenotypic switching, inflammatory activation, and increased LDL uptake [166]. ABCA1 is a direct target of miR-92a and is a component of the miR-17-92 cluster [169]. ABCA1 is an important participant in reverse cholesterol transport, a process by which cellular cholesterol is exported to an extracellular acceptor with the formation of LDL. Importantly, excessive cholesterol accumulation due to reduced expression or functional activity of ABCA1 leads to their proinflammatory activation through several mechanisms. These data enhance the understanding of the role of miR-92a as an important participant in providing various mechanisms of arterial homeostasis [166].

Endothelial-mesenchymal transition (EndMT) is a process of cell differentiation in which endothelial cells acquire mesenchymal properties. EndMT is associated with the development of atherosclerosis and can cause a number of phenotypic changes in endothelial cells, including their dysfunction as well as plaque formation [170]. Unidirectional laminar flow protects endothelial cells from EndMT, whereas impaired flow promotes EndMT formation. Laminar flow has been shown to induce the expression of TN-X (tenascin-X) extracellular matrix protein in mouse and human endothelial cells via the transcription factor KLF4. In turn, TN-X inhibits the activity of TGF-β and EndMT [171]. In addition, PFKFB3 is considered to be a critical factor of EndMT [172]. These data enhance the understanding of the role of KLF4 in EndMT regulation. KLF2 has also been shown to inhibit TGF-beta signaling through the induction of inhibitory Smad7 and attenuation of AP-1 activity [173].

KLF2 is also considered as a transcriptional regulator of endothelial thrombotic function [174]. KLF2 overexpression increased clotting time as well as blood flow velocity in basal and inflammatory conditions. This is due to KLF2-mediated induction of thrombomodulin (TM) and eNOS expression and decreased expression of plasminogen activator inhibitor-1 (PAI-1) [174]. KLF4 also protects against atherothrombosis in mice by inducing an anti-adhesive and anti-thrombotic state of endothelial cells [175].

Laminar flow has also been shown to inhibit vascular calcification by inhibiting endothelial bone morphogenetic protein (BMP)/SMAD1/5 signaling via KLF2 [176]. Vascular calcification plays an important role in the pathogenesis of atherosclerosis; thus, the role of KLF2 is of clinical interest.

Thus, KLF2 and KLF4 play crucial roles in the molecular mechanisms of atherosclerosis, regulating multiple pathways involved in endothelial cell function and other vascular wall and blood flow cells, as well as in inflammation and lipid metabolism. The protective effects of KLF2 and KLF4 in atherosclerosis make them an attractive target for the development of new treatments for cardiovascular diseases.

## 4. Conclusions

Atherosclerosis is a complex, chronic inflammatory disease that is the leading cause of cardiovascular disease and the main cause of death worldwide. The pathogenesis of atherosclerosis involves various cellular and molecular mechanisms, including endothelial dysfunction, immune and inflammatory responses, impaired lipid metabolism, and oxidative stress.

Endothelial dysfunction, characterized by impaired nitric oxide bioavailability, is considered to be one of the key early stages of atherogenesis. Endothelial cells play an important role in the regulation of vascular hemodynamics, immune defense, and other cellular functions, and their dysfunction contributes to the onset and progression of atherosclerosis. Recent advances in understanding the cellular and molecular mechanisms of atherosclerosis have revealed an important place for the KLF family of transcription factors, which regulate various cellular functions and pathways associated with atherogenesis.

Thus, KLFs are important regulators of atherogenesis, and understanding their functions in the development and progression of atherosclerosis may provide new therapeutic targets for the prevention and treatment of cardiovascular diseases.

## Figures and Tables

**Table 1:** Participation of Kruppel-like factors in various biological processes.

KLFs	Names	Biological Process
KLF1	Krueppel-like factor 1, Kruppel like factor 1, erythroid Kruppel-like factor, erythroid krueppel-like transcription factor, erythroid-specific transcription factor EKLF.	myeloid cell differentiation, erythrocyte differentiation, regulation of gene expression, regulation of transcription by RNA polymerase II
KLF2	Krueppel-like factor 2, Kruppel-like factor 2, Kruppel-like factor 2 (lung), Kruppel-like factor LKLF, lung Kruppel-like factor, lung Kruppel-like zinc finger transcription factor, lung krueppel-like factor.	cell morphogenesis, cellular response to laminar fluid shear stress, positive regulation of nitric oxide biosynthetic process, vasodilation, cellular response to interleukin-1, cellular response to tumor necrosis factor, negative regulation of interleukin-6 production.
KLF3	Krueppel-like factor 3, CACCC-box-binding protein BKLF, Kruppel-like factor 3, Kruppel-like factor 3 (basic) TEF-2, basic Kruppel-like factor, basic krueppel-like factor, basic kruppel-like factor. transcript ch138	regulation of transcription by RNA polymerase II, regulation of transcription, DNA-templated.
KLF4	Krueppel-like factor 4, Kruppel-like factor 4, Kruppel-like factor 4 (gut), endothelial Kruppel-like zinc finger protein, epithelial zinc finger protein EZF, gut Kruppel-like factor, gut-enriched krueppel-like factor.	cellular response to laminar fluid shear stress, canonical Wnt signaling pathway, cellular response to growth factor stimulus, negative regulation of angiogenesis, negative regulation of cell population proliferation, negative regulation of inflammatory response, negative regulation of leukocyte adhesion to arterial endothelial cell, negative regulation of response to cytokine stimulus, negative regulation of NF-kappaB transcription factor activity, negative regulation of interleukin-8 production
KLF5	Krueppel-like factor 5, intestinal Kruppel-like factor, BTE-binding protein 2, GC box binding protein 2, Kruppel like factor 5, Kruppel-like factor 5 (intestinal), basic transcription element binding protein 2, colon krueppel-like factor, colon kruppel-like factor, epididymis secretory sperm binding protein, intestinal-enriched krueppel-like factor, intestinal-enriched kruppel-like factor, transcription factor BTEB2.	Angiogenesis, cell-cell signaling via exosome, positive regulation of fat cell differentiation
KLF6	Krueppel-like factor 6, B-cell-derived protein 1, GC-rich binding factor, GC-rich sites-binding factor GBF, Kruppel-like factor 6, Kruppel-like zinc finger protein Zf9, core promoter element binding protein, proto-oncogene BCD1, protooncogene B-cell derived 1, suppression of tumorigenicity 12 (prostate), suppressor of tumorigenicity 12 protein, transcription factor Zf9.	B cell differentiation, lymphocyte differentiation, B cell activation, positive regulation of DNA-templated transcription
KLF7	Krueppel-like factor 7, Kruppel-like factor 7, Kruppel-like factor 7 (ubiquitous), ubiquitous Kruppel-like factor, ubiquitous Kruppel-like transcription factor.	regulation of adipose tissue development, negative regulation of insulin secretion, regulation of insulin secretion involved in cellular response to glucose stimulus, glucose homeostasis, positive regulation of transcription by RNA polymerase II
KLF8	Krueppel-like factor 8, Kruppel-like factor 8, basic krueppel-like factor 3, basic kruppel-like factor 3, zinc finger protein 741.	negative regulation of transcription by RNA polymerase II, negative regulation of transcription, DNA-templated
KLF9	Krueppel-like factor 9, BTE-binding protein 1, GC-box-binding protein 1, Kruppel-like factor 9, basic transcription element-binding protein 1, transcription factor BTEB1.	cellular response to thyroid hormone stimulus, cellular response to glucocorticoid stimulus, cellular response to ketone, cellular response to hormone stimulus, circadian rhythm.
KLF10	Krueppel-like factor 10, Kruppel-like factor 10, TGFB-inducible early growth response protein 1, early growth response-alpha, transforming growth factor-beta-inducible early growth response protein 1, zinc finger transcription factor TIEG.	cellular response to nutrient levels, negative regulation of cell population proliferation, positive regulation of transcription by RNA polymerase II, regulation of cell population proliferation, cell-cell signaling, circadian rhythm, cellular response to starvation
KLF11	Krueppel-like factor 11, Kruppel-like factor 11, TGFB-inducible early growth response protein 2, TIEG-2, transforming growth factor-beta-inducible early growth response protein 2.	apoptotic process, cellular response to peptide, negative regulation of cell population proliferation, regulation of transcription involved in G1/S transition of mitotic cell cycle, negative regulation of transcription, DNA-templated
KLF12	Krueppel-like factor 12, AP-2 repressor, AP-2rep transcription factor, KLF12 zinc finger transcriptional repressor, Kruppel-like factor 12, transcriptional repressor AP-2rep.	negative regulation of transcription by RNA polymerase II, negative regulation of transcription, DNA-templated
KLF13	Krueppel-like factor 13, BTE-binding protein 3, Kruppel-like factor 13, RANTES factor of late activated T lymphocytes-1, basic transcription element binding protein 3, novel Sp1-like zinc finger transcription factor 1, transcription factor BTEB3, transcription factor NSLP1.	transcription, DNA-templated, transcription by RNA polymerase II, negative regulation of cell population proliferation, negative regulation of erythrocyte differentiation
KLF14	Krueppel-like factor 14, BTE-binding protein 5, Kruppel-like factor 14, basic transcription element-binding protein 5, transcription factor BTEB5.	positive regulation of sphingolipid-mediated signaling pathway, positive regulation of transcription by RNA polymerase II, regulation of transcription by RNA polymerase II
KLF15	Krueppel-like factor 15, Kruppel-like factor 15, kidney-enriched Kruppel-like factor, kidney-enriched krueppel-like factor.	cardiac muscle hypertrophy in response to stress, cellular glucose homeostasis, cellular response to peptide, positive regulation of glucose import, regulation of Wnt signaling pathway, response to insulin
KLF16	Krueppel-like factor 16, BTE-binding protein 4, Kruppel-like factor 16, basic transcription element binding protein 4, dopamine receptor regulating factor, novel Sp1-like zinc finger transcription factor 2, transcription factor BTEB4, transcription factor NSLP2.	dopamine receptor signaling pathway, regulation of transcription by RNA polymerase II
KLF17	Krueppel-like factor 17, Kruppel-like factor 17, novel zinc-finger protein, zinc finger protein 393.	regulation of transcription by RNA polymerase II
KLF18	Kruppel-like factor 18, KLF pseudogene, Kruppel-like factor 18.	regulation of transcription by RNA polymerase II
